# The X-linked Retinitis Pigmentosa protein RP2 facilitates traffic of cilia target proteins

**DOI:** 10.1186/2046-2530-1-S1-P45

**Published:** 2012-11-16

**Authors:** N Schwarz, TV Novoselova, R Wait, AJ Hardcastle, ME Cheetham

**Affiliations:** 1 UCL, Institute of Ophthalmology, UK; 2 ORBIT, UK; 3 Kennedy Institute of Rheumatology Division, Imperial College London, UK

## 

Photoreceptors are specialized ciliated sensory neurons and aberrant traffic of proteins to the outer segment causes photoreceptor cell death. RP2 is a GTPase activating protein (GAP) for the small GTPase Arl3 and both proteins facilitate protein trafficking to primary cilia. We used GST-RP2 pull down from retinal lysates and identified the Gβ subunit of transducin (Gβ1) as a novel RP2 interacting protein. RP2 competes with Gγ1 for Gβ1 binding and does not interact with the Gβ:Gγ heterodimer. In SK-N-SH cells, overexpression of Gβ1 resulted in the cytoplasmic accumulation of the protein, whereas co-expression of Gβ1 with either RP2 or Gγ1 restored membrane association of Gβ1. Depletion of RP2 in ARPE19 cells by siRNA resulted in a shift of Gβ1 from the membrane to the cytosol, confirming that RP2 facilitates the membrane association of Gβ1. This shift in Gβ1 localization was rescued by Gγ1 overexpression. Membrane targeting of Gβ1 required RP2 N-terminal myristoylation and occurs via the co-factor C (TBCC) homology domain. The interaction was disrupted by the pathogenic RP2 mutation R118H, which blocks Arl3 GAP activity. Arl3-Q71L competed with Gβ1 for RP2 binding suggesting that RP2 GAP activity on Arl3 would release Gβ1. RP2 stimulated the association of Gβ1 with Rab11, an important GTPase for post-Golgi vesicle trafficking of photoreceptor proteins. Collectively our data support a role for RP2 in facilitating membrane association and traffic of Gβ1. Combined with other recent evidence, this suggests that RP2 may co-operate with Arl3 and its effectors in cilia associated trafficking of G proteins.

